# Mr. Jenkinson, on Rest in Phthisis Pulmonalis

**Published:** 1809-08

**Authors:** John Jenkinson

**Affiliations:** Oxford


THE
Medical and Phyfical Journal.

VOL. XXII.] AUGUST, 1809. [NO. 126.

*Printed for R. PHILLIPS, by W. Thorne, Red Lion Court, Fleet Street, London.*

### *To the Editors of the Medical and Phyfical Journal.*

GENTLEMEN,

EITHER your Readers have not deemed my inquiries concerning the employment of rest, in the first stage of Phthisis Pulmonalis worthy of their attention, or they are not possessed of such information as I requested from them upon the subject. When it is considered too that the best authors who have treated on the means of resisting this fatal disease have not thrown out a single hint on the utility of rest, as an auxiliary in any of its stages, I ought not to wonder, however mortifying it may be, if none of your Correspondents have noticed my address, through the channel of the Medical and Physical Journal for March last. This shall not however deter me from keeping my promise by asking the favor of three or four of your pages, for the purpose of recommending the trial of local and general repose, under the circumstances before mentioned. The inadequacy of our present modes of arresting the progress of consumption is undeniable; but not more so, I fear, than the rapid increase of its ravages in this country: if, then, there is one disease that calls more loudly than another for the introduction of additional methods of relief, surely it is pulmonary consumption.

I have no intention of dwelling upon the causes, the history, or the nature of the disease in question; there is no dearth of information upon any of those points; and it will be sufficient for my purpose to state, that *in its origin* it is supposed to consist, from whatever cause it may proceed, in the excessive or irritated action of some one or more sets of the vessels subservient to the functions of the affected viscus; that is to say, in a peculiar excitement or inflammation, tending to the formation of what are denominated tubercles of the lungs. Accordingly, the most promising remedies are generally held to be such as contribute most effectually to restrain the circulation of the blood within moderate limits, both in the system at large, and more especially in the lungs themselves. Into this, and the counter irritation of some neighbouring or connected part or parts, most of the indications of cure which maintain their credit (for the first stage of the disorder) will be found to resolve themselves: but it would be difficult to shew how exercise in the open air, particularly in our variable climate, can at any season of the year agree with such a treatment. Sydenham, it is true, strongly recommends riding on horseback in consumption, without specifying the circumstances under which it is advantageous;* and he is too accurate an observer to have mentioned its utility without good grounds. In the advanced periods of phthisis, when suppuration has become evident, and the debility great, no doubt, every thing which cheers the patient's spirits, and helps to support his strength, must contribute in the same proportion to prolong his existence: but surely it must be prejudicial at an earlier period. In the inflammation, irritation, or excitement, (call it which you please) whether phlegmonous or peculiar of any other part, exercise would be one of the last means which a rational man would wish to resort to; and there appears not much more propriety in it at the beginning of consumption, than there would in an attack of pneumonia or enteritis. Yet nothing is more common than to see a consumptive patient in the first stage, who is actually under treatment intended to reduce the pulse at the very time, left almost as much at liberty in this and other respects as any of the healthy members of the family; just as if we were yet ignorant that the pulse is much quicker in an upright posture and a talkative mood, than it is in a state of repose and silence.

We all know that in an active hæmorrhage from the lungs, which is frequently an early symptom of phthisis pulmonalis, speech and motion are most strictly and properly forbidden; but because, in the great majority of consumptive cases, the arteries of the lungs are able to sustain the increased force or celerity with which the blood is transmitted

---

* I have since found that I trusted too much to my memory in asserting this of Sydenham, *since he distinctly points out two of the later symptoms as those relieved by riding on horseback*; and thus it appears that the benefit he had seen derived from it must have been at an advanced period of the disease, as I had previously supposed. Hoffman objects in express terms to the use either of a horse or a carriage in the *beginning* of phthisis. Wallis's Sydenham, vol. II. p. 155, Ed. 1788. Hoffman Opera, tom. 3, p. 294.

mitted through them, without bursting, these means are altogether neglected; although, except this accidental occurrence, the circumstances of the patient are the same, and the circulation of the part is probably as much out of bounds in the one case as in the other. Nor is the increased action of the heart and arteries the only injurious effects of exercise and conversation in these cases; the hurried respiration which necessarily results from them, affords the very means by which this rapid circulation is carried on, viz. the equally rapid expansion of the lungs, to say nothing of the pain and fatigue which is sooner or later occasioned to persons thus afflicted by the exertion. The extremities are subject to as great a variety of inflammations as the lungs, though not perhaps exactly of the same kind, and it would be reckoned proposterous to allow a patient suffering from the very slightest of them to swing his arm, or walk about, during the cure; although the worst that can happen in such cases is for the most part curable.

Every other plan likely to assist us in subduing this bane of our country has been tried in vain, and why should not complete bodily rest, combined with every possible quiescence of the excited parts be at least attempted? It would certainly be too much to say that it would supply the defect altogether; but if reasoning from analogy be at all applicable upon this occasion, it is not too much to hope that, *employed in time*, it might prove one step towards it. Besides the great advantage of agreeing with the most rational indications of cure already established, it possesses in a high degree that of assisting and facilitating the execution of our other means of relief. If you are seeking to keep down your patient's pulse by sedatives or evacuants, can you do better than confine him to his bed or couch, in a posture which of itself will moderate the circulation as much as the most powerful remedy, without farther diminishing the strength? If you are trusting to the effects of a regular and congenial temperature, by adopting this plan, you have your patient strictly confined to his own apartments. Are you placing your dependence upon particular articles of diet; in this way the sick is preserved from every temptation to deviate. If you expect to relieve by blisters, issuesor setons, the most effectual method of rendering them tolerable to your patient, is to prevent himfrom irritating them by moving about. If you are hopeless of more than the palliation of symptoms, in a recumbent postureconstantly observed, you will find his pain of the breast or side, and his harrassing cough comparatively quiet.

From the circumstance of these two symptoms, being often most troublesome upon first going to bed and rising in a morning, some will be inclined to deny that they are relieved in the manner I have mentioned; but it should be recollected, that it is not either one posture or the other which ought to be made accountable for this distress, but the *change* from an upright to a recumbent, and from a recumbent to an upright posture.

Without attempting to anticipate all the objections, to which the use of rest and silence in early consumption may be liable; let me advert to such of them as lie pretty much upon the surface. In the first place, to the difficulty of enforcing what will appear so irksome as this kind of restraint for two or three months together, I am aware that there are many. consumptive patients, who must follow their usual occupations, from necessity, till long after it is too late for them to benefit by any change; and others, who cannot be made to feel their danger at any period; but, after these deductions, many interesting and valuable subjects of this dreadful malady remain, who would not resist the intreaties of their relatives, or the advice of their medical attendant. Such consumptive affections as evidently betray a strumous origin, and which, I will not affect to deny, form a very considerable proportion of the whole, may prove beyond the reach of art, at a very early period of attack; but I think there can be very little doubt that the mischief is first *set to work*, and afterwards *kept up*, by the same causes and in the same manner as in other habits, and of course that the same means are required, though with less prospect of success. But this unfortunate state of things, demands from us attention and not indifference, activity and not despair. I have now done with this subject, at least for the present; but I shall be obliged to any one who pursues it further, in the way either of assistance or correction; and shall be very ready to give any further explanation that may seem to be required of me, or to retract any thing on being shewn in what my error consists.

At the same time, I wish to offer two or three remarks concerning Digitalis: We all recollect how very greatly it was extolled in consumption a few years ago. No doubts were admitted of its efficacy, or utility at least, in *any* stage of the disorder. We have now time to cool upon it, and to see the medical world divided into several parties about this important plant: some prescribe it in inflammatory diseases, and consequently at the *beginning*

of of consumption; some in those of debility,* and consequently at the latter end of it; while some employ it in neither, and, what is most extraordinary, some in both. Of course, some believe it to be an *universal* sedative,† and others an *universal* stimulant. It is clear from all this, that we have still to learn, what are really the effects of this once fashionable medicine? Perhaps, the difficulty may be solved in something like the following manner: A quick pulse occurs in two opposite states of the body; namely, in extreme exhaustion and extreme excitement. Now the quickness of debility is, generally speaking, reduced by those medicines which have obtained the title of stimulants; and the quickness of inflammation by those called sedatives; what accelerates the one reduces the other, and vice versa; consequently, the same medicine has opposite effects in opposite states of the body, or, in other words, is by turns both a stimulant and a sedative. If this be the case, it is the *condition of the patient* which determines, in a great measure, the effect of what he swallows; and the confusion which I have been speaking of, probably, arises from this distinction having been too often disregarded. If so, we need not be much at a loss to account for most of the contradictions, with which makers of experiments upon articles of the materia medica, have saluted each other; since it is not only requisite for them to understand each other's terms, but also to choose subjects as nearly as possible of the same strength and temperament.

Whether it is the quickness of inflammation, or that of debility, which digitalis has the power of decreasing, although I believe it to possess the latter only, and therefore to be fit only for advanced consumption, it would not become me to assume the office of declaring: I merely contend that it cannot do *both,* and that unless the two states I have alluded to, be kept separately in view whenever the point is contested, the disputants will be apt to fall into the situation of the Knights who came to blows about the colour of the Statue, which appeared black or white, according to the ground occupied by the respective Champions.

*Oxford, July* 12, 1809. JOHN JENKINSON.

---

* This party have the early practice of Drs. Withering and Darwin, which preceded all theory upon the subject, on their side.

† Dr. Ferriar, for instance, says—"If any man had expressed an opinion a few years ago, that we should discover a medicine capable of reducing the pulse, without danger, from 120 in a minute to 75 or 80, *at the will of the practitioner,* he would have been ridiculed as a visionary; yet such is the power of digitalis under proper management."

